# Unsupervised clustering of serum lipase activity in cats: a data-driven approach to correlate clinical, laboratory, and ultrasonographic findings

**DOI:** 10.1093/jvimsj/aalag072

**Published:** 2026-04-21

**Authors:** Vanessa Hotz, Daniel Brugger, Peter H Kook

**Affiliations:** Clinic for Small Animal Internal Medicine, Vetsuisse Faculty, University of Zurich, Zurich 8057, Switzerland; Department of Agricultural Sciences, Faculty of Agriculture and Forestry, University of Helsinki, P.O. Box 27, FI-00014 Helsinki, Finland; Clinic for Small Animal Internal Medicine, Vetsuisse Faculty, University of Zurich, Zurich 8057, Switzerland

**Keywords:** clinical signs, laboratory findings, cats, lipase activity, pancreatitis, ultrasonography

## Abstract

**Background:**

Serum lipase activity has not been compared with ultrasonographic and clinical findings in cats.

**Hypothesis/Objectives:**

Interpret serum lipase activity in sick cats using unsupervised, data-driven clustering.

**Animals:**

Five hundred sixty-three hospitalized client-owned cats with serum lipase activity > 30 U/L (reference intervals, 8-26 U/L)

**Methods:**

Retrospective study. Unsupervised k-means clustering of serum lipase activity (*k* = 3, chosen by elbow and silhouette diagnostics) grouped cats into 3 ranges. Least squares mean (LSmean) lipase values were: cluster 1: 53 U/L (range, 30-125 U/L); cluster 2: 229 U/L (range, 147-411 U/L); and cluster 3: 778 U/L (range, 441-1613 U/L). Clinical (*n* = 563), laboratory (*n* = 563), and ultrasonographic (*n* = 318) findings were compared across clusters using general linear models and Fisher’s exact tests with multiplicity correction (Bonferroni).

**Results:**

Ultrasonographic diagnosis of pancreatitis (USDx) was significantly (*P* = .0021) more common in cluster 2 (64%) compared to cluster 1 (47%). Pancreatic enlargement (59%), hypoechogenicity (56%), and hyperechoic mesentery (49%) were significantly more common in cluster 2 compared to cluster 1, but 40%-50% of cluster 2 cats and 25%-66% of cluster 3 cats did not have these US abnormalities. Only lethargy was significantly more common in cluster 2 cats. Significant differences in laboratory values were found for urea (highest in cluster 3), triglycerides (highest in cluster 2), and protein (lowest in cluster 3).

**Conclusions and clinical importance:**

Despite marked hyperlipasemia, approximately 50% of cats have no ultrasonographic pancreatic abnormalities or changes insufficient for an USDx. Future routine pancreatic size assessment might help detect subtle changes. Abdominal pain is not significantly more common at higher lipase activities.

## Introduction

Pancreatitis is a common histopathological finding in feline necropsy studies,^1–3^ In practice, pancreatitis is diagnosed clinically based on a combination of clinical, laboratory, and imaging findings.^4–6^ Determination of serum lipase as an activity using the substrate DGGR (1,2-o-dilauryl-rac-glycero-3-glutaric acid-(6′-methylresorufin) ester) or a concentration using an immunoassay (pancreatic lipase immunoreactivity, PLI) is considered the most important laboratory test to diagnose pancreatitis in cats.^2,6–10^ Both assays correlate highly (correlation coefficients between 0.82 and 0.98),^2,10–12^ and do not differ when compared with pancreatic histology or ultrasonographic (US) examination. However, agreement between both lipase assays and US is only fair.^2,10^ The clinical relevance of hyperlipasemia in cats is often unclear. Sick cats tend to have nonspecific clinical signs (ie, lethargy, inappetence), and cats with histologically confirmed acute or chronic pancreatitis do not differ clinically.^13^ Reference intervals (RI) have been determined for clinically healthy cats,^9,14^ but it remains unclear at what lipase activity clinically relevant pancreatitis (ie, the cat is sick because of pancreatitis) occurs. Consequently, there are no established diagnostic cut-off values for serum lipase concentration and activity for a definitive diagnosis of pancreatitis.^15,16^ Moreover, sick cats almost never have pancreatitis alone^9,16–19^ further complicating the clinician’s assessment. This explains why pancreatic ultrasonography is an important diagnostic component in hyperlipasemic cats, as it can aid in the assessment of pancreatic changes, although the absence of abnormalities does not rule out clinical pancreatitis. Pancreatic US findings have so far only been compared with serum lipase activity and PLI concentration values above the respective RI.^10^ Similarly, sensitivities and specificities or prognostic values of pancreatic US abnormalities have only been compared to PLI concentration RI cutoffs^17–20^ a limit that has since been revised.^15^ It is unknown whether pancreatic US abnormalities can be detected more frequently at higher lipase activities. The same applies to clinical signs and laboratory values. This is why we aimed to use an unsupervised clustering approach to investigate whether clinical findings differ across varying degrees of lipase activity in cats. This methodology allows data to reveal natural groupings without predefined diagnoses.^21^ Transformation of a continuous biomarker (serum lipase activity) into a categorical framework allows to assess how clinical characteristics distribute across subgroups.

Our objective was to investigate whether lipase clusters differ in terms of clinical signs, laboratory and US changes. We hypothesized that cats with higher serum lipase activity will have greater severity in ultrasonographic changes.

## Materials and methods

### Case selection and data collection

Cases were identified by searching medical records at the authors’ institution between October 2017 and January 2020. Inclusion criteria were hospitalization and inpatient treatment and a Roche Colorimetric Lipase (LIPC) DGGR-based serum lipase activity result > 30 U/L (RI, 8-26 U/L). When a cat met these criteria more than once, only data from the first hospitalization were recorded. Data collection encompassed signalment (age, weight), clinical, laboratory (CBC, serum biochemistry profile), US findings at presentation, as well as presence of comorbidities. Duration of disease before presentation, duration of hospitalization (DoH) and duration of intensive care unit stay (DoIPS) were also recorded. The comparison of clinical signs and laboratory variables was conducted across all cats in the clusters, irrespective of whether an US was performed. Clinical signs were obtained from medical history and initial examination reports, focusing on the presence or absence of vomiting, diarrhea, lethargy, anorexia, painful abdomen, polyuria, polydipsia (Pu/Pd), and weight loss. Presence or absence of pancreatic US findings (enlargement, hypoechogenicity, hyperechogenicity, mixed echogenicity, hyperechoic mesentery, peripancreatic fluid, and a final US diagnosis of pancreatitis (USDx) were recorded. Changes of the hepatobiliary system (presence of gallbladder sludge, hepatomegaly, hepatic hypoechogenicity, hepatic hyperechogenicity, mixed echogenicity), and gastrointestinal tract (lamina muscularis thickening, mesenteric lymphadenopathy, gastrointestinal masses) abnormalities were also recorded. The US pancreatic appearance was assessed using a modified ultrasonographic pancreatic assessment severity score (UPASS^22^) ranging from 0 to maximally 6 (Table 1). Mesenteric echogenicity was graded as either normal (=0) or hyperechoic (=1).

**Table 1:** Modified US pancreatic assessment severity score (UPASS) from Cridge et al.^21^

Component of the UPASS	Assigned score		
	0	1	2
**Pancreatic size**	Normal	Enlarged	
**Pancreatic echogenicity**	Normal	Hyperechoic	Hypoechoic
**Pancreatic echotexture**	Homogenous	Heterogenous	
**Echogenicity of surrounding mesentery**	Normal	Hyperechoic	
**Peripancreatic fluid**	No	Yes	

This table outlines the criteria and corresponding scores used in the US Pancreatic Assessment Scoring System (UPASS). Each component of the pancreas (size, echogenicity, echotexture, echogenicity of surrounding mesentery, and peripancreatic fluid) is evaluated and assigned a score (0, 1, or 2) based on the observed characteristics.

All examinations were performed by board-certified diagnostic imagers and residents under direct supervision of a specialist using the GE HealthCare US device LOGIQ TM E10 Series. Medical diagnoses of cats were extracted from referral letters and medical records and then categorized by organ systems. These categories included gastrointestinal, nephrology/urology, hepatobiliary, endocrine, cardiac, respiratory, musculoskeletal, nervous system, and ophthalmologic diseases. Data collection was performed as previously described.^16^ Institutional ethical approval was not required because this was a retrospective study and all clients had given signed consent for use of their cat’s clinical information in research.

### Statistical analyses

Statistical analyses are detailed in Appendix section.

## Results

### Cats and lipase clusters

**Figure 1:**
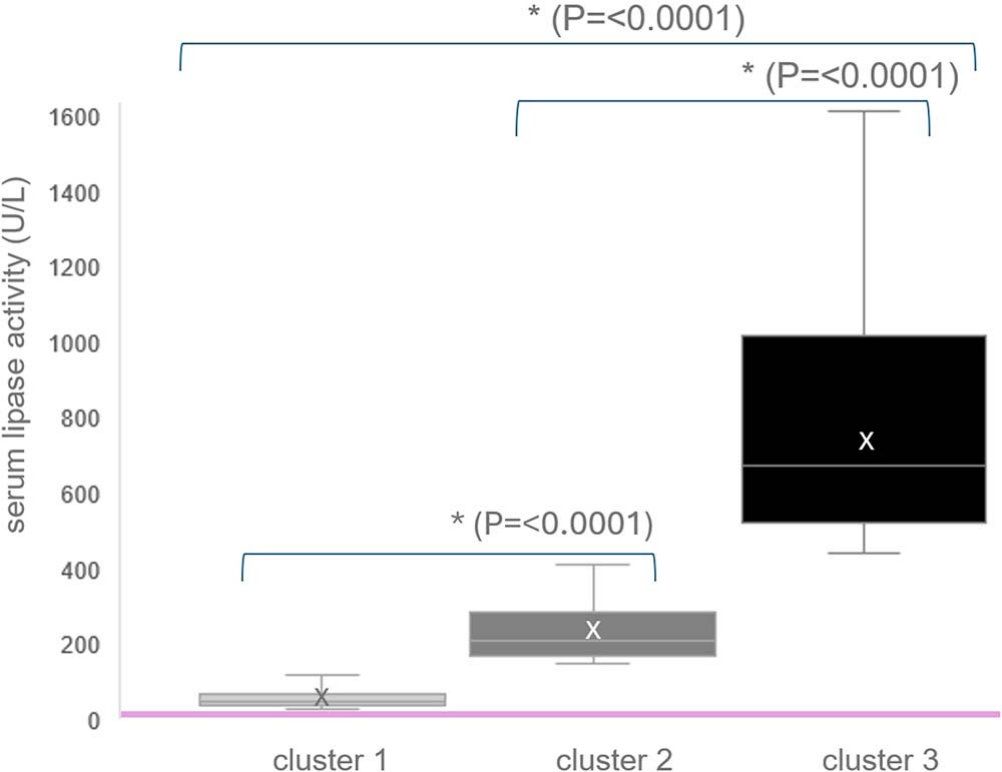
Distribution of serum lipase activities (U/L) for each cluster. The boxes represent the interquartile range (IQR), the horizontal line within the box indicates the median, and the “x” denotes the LSmean lipase value. The asterisk (*) denotes a statistically significant difference. Whiskers extend to 1.5 times the IQR from the upper and lower quartiles, respectively. Note that the RI of 8-26 U/L for lipase activity is additionally indicated.

The study cohort comprised 563 cats (324 males, 239 females; including 308 castrated males and 222 spayed females). Using unsupervised k-means clustering, cats were categorized into 3 distinct clusters based on the magnitude of serum lipase activity: cluster 1 (*n* = 494, 88%), cluster 2 (*n* = 54, 9%), and cluster 3 (*n* = 15, 3%). LSmean lipase values and ranges were: cluster 1: 53 U/L (range, 30-125 U/L); cluster 2: 229 U/L (range, 147-411 U/L); and cluster 3: 778 U/L (range, 441-1613 U/L) (Figure 1). Within cluster 1, 63/494 cats (13%) had lipase values exceeding 3 times the study inclusion threshold (> 90 U/L, inclusion criterion ≥30 U/L).

LSmeans of age and body weight were not different between clusters (LSmeans age: 8.85, 8.18, and 9.37 years; LSmeans body weight: 4.26, 4.15, and 4.29 kg). Additionally, the duration of disease before presentation, DoH, or DoIPS were not different between clusters.

### Distribution of US findings

US examinations were performed more frequently in cats in cluster 3 (12/15, 80%) and cluster 2 (39/54, 72%) compared to cluster 1 (267/494, 54%). This was significantly different between cluster 1 and cluster 2 (*P* = .0136). Distribution of specific US findings within each cluster is detailed in Table 2. An USDx was significantly more common in cluster 2 compared to cluster 1 (*P* = .0021), but one-third of cats in cluster 2 and 7 out of 12 of cats in cluster 3 did not have an USDx. The UPASS median scores and ranges were 2 (0-6) for cluster 1, 3 (0-6) for cluster 2, and 2.5 (0-6) for cluster 3, with cluster 2 exhibiting a significantly higher median than cluster 1 (*P* = .0002).

**Table 2:** US findings across the study clusters.

Ultrasonographic findings		cluster 1(*n* = 267) Lipase: LSmean 53 U/L (range, 30-125 U/L)	cluster 2(*n* = 39) Lipase: LSmean 229 U/L (range, 147-411 U/L)	cluster 3(*n* = 12) Lipase: LSmean 778 U/L (range, 441-1613 U/L)
**Pancreas**	USDx	125^a^ (47%)	25^bc^ (64%)	5^ac^ (42%)
	Enlargement	91^a^ (34%)	23^b^ (59%)	9^b^ (75%)
	Hypoechogenicity	89^a^ (33%)	22^bc^ (56%)	5^ac^ (42%)
	Mixed echogenicity	66^a^ (25%)	18^bc^ (46%)	5^ac^ (42%)
	Hyperechoic mesentery	63^a^ (24%)	19^bc^ (49%)	4^ac^ (33%)
	Hyperechogenicity	42 (16%)	7 (18%)	1 (8%)
	Peripancreatic fluid	19 (7%)	5 (13%)	3 (25%)
**Hepato-biliary system**	Enlargement	104 (39%)	13 (33%)	5 (42%)
	Hypoechogenicity	42 (16%)	2 (5%)	0 (0%)
	Mixed echogenicity	48 (18%)	7 (18%)	4 (33%)
	Hyperechogenicity	118 (44%)	16 (41%)	6 (50%)
	Gallbladder sludge	60 (22%)	10 (26%)	3 (25%)
**Intestines**	L. muscularis thickening	114 (43%)	16 (41%)	2 (17%)
	Mesenteric lymphadenopathy	101 (38%)	18 (46%)	3 (25%)
	Intestinal mass	10^a^ (4%)	1^ab^ (3%)	3^b^ (25%)

Fisher’s exact test was applied to test for differences in frequencies of ultrasonographic findings between groups. For each variable, an overall 3 × 2 test was followed by 3 pairwise comparisons between clusters. Because of these 3 pre-specified comparisons, a Bonferroni correction was applied and the Type I error threshold adjusted to *P* ≤ .0167. Frequencies between groups not sharing a common superscript are considered significantly different at *P* ≤ .0167. See Table S3 for precise between-Cluster *P*-values.

Pancreatic enlargement was significantly more common in clusters 3 (*P* = .01) and 2 (*P* = .0041) compared to cluster 1, but over 40% of cats in cluster 2 and 3 out of 12 of cats in cluster 3 had no evidence of pancreatic enlargement (Table 2). Cluster 2 exhibited the highest frequency of pancreatic hypoechogenicity (*P* = .0071 vs. cluster 1) and mixed echogenicity (*P* = .0072 vs. cluster 1), again over 40% and nearly 60% of cats in cluster 2 and 3, respectively had no evidence of pancreatic hypoechogenicity and mixed echogenicity. A hyperechoic mesentery was also more prevalent in cluster 2 (*P* = .0017 vs. cluster 1) with 48% of cases having an hyperechoic mesentery, two-thirds of cluster 3 cats did not have a hyperechoic mesentery. US changes of the liver and gallbladder did not differ between clusters (Table 2), When comparing US changes of the intestines between clusters, only the presence of an intestinal mass varied significantly between clusters (*P* = .0158), although this finding was rare across all clusters (Table 2).

### Distribution of clinical signs

The frequency of observed clinical signs across the 3 clusters is given in Table 3. Among the recorded signs, only lethargy differed significantly in frequency between groups (*P* = .014). The median number of clinical signs was 2 (range, 0-7) for cluster 1, 3 (range, 0-6) for cluster 2, and 3 (range, 0-5) for cluster 3, with no significant differences between clusters.

**Table 3:** Frequency of clinical signs across clusters.

Clinical signs	cluster 1 (*n* = 494) Lipase: LSmean 53 U/L (range, 30-125 U/L)	cluster 2 (*n* = 54) Lipase: LSmean 229 U/L (range, 147-411 U/L)	cluster 3 (*n* = 15) Lipase: LSmean 778 U/L (range, 441-1613 U/L)
**Lethargy**	253^a^ (51%)	38^b,c^ (70%)	10^a,c^ (67%)
**Anorexia**	250 (51%)	32 (59%)	10 (67%)
**Vomiting**	190 (38%)	29 (54%)	7 (47%)
**Icterus**	116 (23%)	1 (2%)	0 (0%)
**Weight loss**	109 (22%)	16 (30%)	5 (33%)
**Pu/Pd**	77 (16%)	9 (17%)	4 (27%)
**Diarrhea**	74 (15%)	10 (19%)	1 (7%)
**Painful abdomen**	51 (10%)	7 (13%)	4 (27%)
**Fever**	26 (5%)	4 (7%)	1 (7%)
**Bloody diarrhea**	9 (2%)	0 (0%)	0 (0%)
**Bloody vomiting**	7 (1%)	1 (2%)	1 (7%)

Fisher’s exact test was applied to test for differences in frequencies of clinical signs between groups. For each clinical sign, an overall 3 × 2 test was followed by 3 pairwise comparisons between clusters. A Bonferroni correction was applied for these 3 comparisons, yielding an adjusted significance threshold of *P* ≤ .0167. Frequencies between groups not sharing a common superscript are considered significantly different at *P* ≤ .0167. See Table S3 for precise between-Cluster *P*-values.

### Distribution of routine laboratory findings

**Figure 2:**
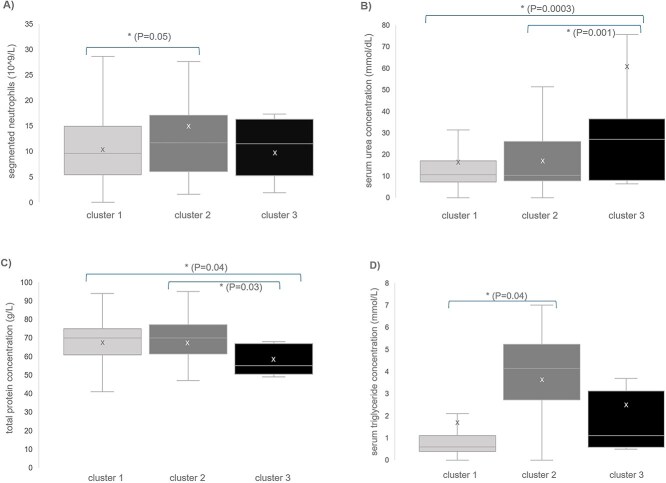
Distribution of selected laboratory variables across clusters. Box plots illustrate the distribution of (A) segmented neutrophils, (B) urea concentration, (C) total protein concentration, and (D) serum triglyceride concentration, stratified by clusters. The boxes represent the interquartile range (IQR), the horizontal line within the box indicates the median, and the “x” denotes the LSmean. The asterisk (*) denotes a statistically significant difference. Whiskers extend to 1.5 times the IQR from the upper and lower quartiles, respectively.

LSmean values and distribution of all variables across clusters are visually represented in Figure 2. Segmented neutrophil counts approached but did not reach statistical significance between clusters (*P* = .054), with higher counts in cluster 2 compared to cluster 1. Serum urea concentrations differed significantly between clusters (*P* = .05), with cluster 3 exhibiting significantly higher urea concentrations compared to both cluster 1 and cluster 2. Serum protein concentrations also differed significantly between clusters (*P* = .03), with cats in cluster 3 showing lower protein concentrations compared to cluster 1 and cluster 2. Serum triglyceride concentrations were significantly different between clusters (*P* = .04), cluster 2 had higher triglyceride concentrations compared to cluster 1. None of the other laboratory variables differed significantly between clusters.

### Comparison of frequency of comorbidities

**Figure 3:**
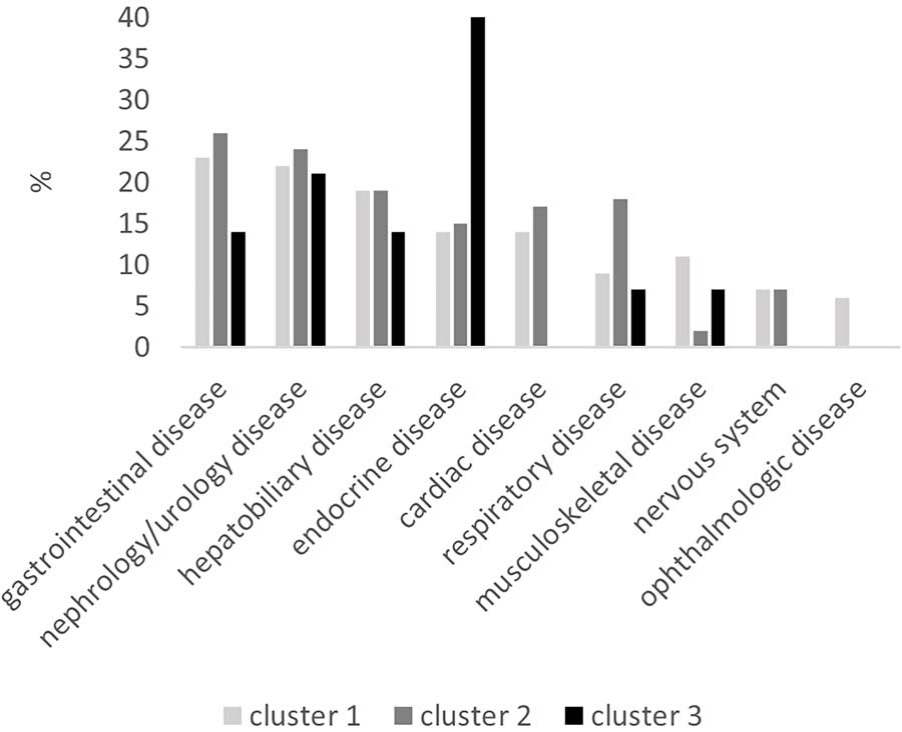
Prevalence (%) of diagnosed comorbidities categorized by organ system for each cluster.

The prevalence of comorbidities was assessed for each cluster, as depicted in Figure 3. There were no significant differences between clusters 1-3. However, a higher numerical occurrence of diabetes mellitus was noted in cluster 3 (6/15) compared to cluster 1 and 2.

## Discussion

This study systematically evaluates clinical variables across different serum lipase activity levels in cats. Because the clinical diagnosis of pancreatitis is inherently uncertain in retrospective studies, we reanalyzed existing data^16^ using a data-driven partitioning strategy based on lipase activities, independent of clinical assessments. We found that despite marked hyperlipasemia, approximately 50% of cats had no pancreatic ultrasonographic abnormalities or non-diagnostic pancreatic changes. Among clinical signs, only lethargy differed between clusters, while serum urea, triglyceride, and total protein concentrations also showed significant differences.

Unsupervised clustering is a machine learning technique designed to assess the data and recognize patterns.^21^ We felt this was particularly useful when investigating sick cats with abnormal lipase activity where disease presentations are variable and poorly understood. To avoid imposing arbitrary cut-offs, the 3 clusters were derived by unsupervised k-means clustering, with the optimal number of clusters (*k* = 3) determined by elbow and silhouette diagnostics (Figure S1). Importantly, additional multivariable models in which serum lipase, age, and body weight were entered as continuous predictors rather than as a cluster factor yielded similar estimates and P-values (data not shown), indicating that our main conclusions are not an artifact of categorizing a single biomarker. For example, although cluster 1 represented the group with the lowest serum lipase activities (mean = 53 U/L, range = 30-125 U/L), 63 cats (13%) within this cluster had values > 3 × the study inclusion threshold (> 90 U/L; inclusion criterion ≥30 U/L). This reflects that cluster assignment was driven by the data distribution rather than by clinical cut-off values.

This highlights a crucial point: cluster boundaries are determined by the dataset’s distribution, not external RI.^23^ Therefore, within the context of this study sample, a lipase value of 125 U/L was considered relatively low, even though a value >  4 × upper RI limit is considered suspicious for pancreatitis at our hospital. Still, the analysis identified distinct groups (clusters 2 and 3) with markedly higher serum lipase activities. We would like to point out here that lipase values around 50 U/L (LSmean cluster 1) are generally considered of limited clinical relevance at our hospital. and are more likely interpreted as indicative of subclinical chronic pancreatitis. Although recent data suggest that cats without clinical suspicion of pancreatitis exhibit higher pancreatic lipase values than previously assumed,^12,15^ it remains unclear to date which lipase cutoff value reliably indicates the presence of pancreatitis. The issue is complicated by the fact that clinically healthy cats can have markedly elevated serum lipase activities and PLI concentration values, also when measured multiple times.^12,15^ Serum lipase activities in cluster 1, although clearly higher compared to RI, are in the range of serum lipase activities in a recent study where clinically healthy cats had median serum lipase activities of 69 U/L (range, 10-283 U/L).^12^

Little has been published on how abnormal lipase can be in cats without clinical signs of pancreatitis. Most likely this is because many hospitals do not have serum lipase activity included in their routine biochemistry profile. Published information is limited to values from healthy cats (RI) and cats with suspected pancreatitis, ie, likely having pancreatic US changes. In contrast at our hospital, serum lipase activity is part of the routine biochemistry profile which naturally provides more information. This means that we also see many cases where lipase results do not necessarily correspond to the supposedly typical US findings of pancreatitis. This provided the opportunity to investigate frequencies of US pancreatic abnormalities in cats categorized by serum lipase activity magnitude. Only about two-thirds of cluster 2 cats (with clearly increased lipase) and 40% of cluster 3 cats (with severely increased lipase) had an USDx. Although this frequency was significantly higher compared to cluster 1 cats, it also means that approximately one-third of cluster 2 cats and 60% of cluster 3 cats had either a normal pancreas or only minor changes on US that were not sufficient for the radiologist to formulate an USDx. This is a novel finding as until now US pancreatic findings in cats have only been compared with upper RI limits of serum lipase activity or PLI concentrations. In fact, most studies do not specify how many cats ultimately received an USDx, but only mention the frequency of the individual pancreatic findings. ^5,7,17,18,20^ Our figures are somewhat higher but still similar to an older study, in which only 35% of cats with histologically confirmed pancreatic necrosis received an USDx.^19^ Improved technology probably accounts for slightly better results for an USDx in clusters 2 and 3 compared to Saunders et al.^19^ But essentially the same questions remain as before, as it was already suggested 23 years ago that more subtle changes in the pancreas should be taken into account in cats compared to dogs.^19^

We assume that many cats in cluster 1 had either chronic pancreatitis or milder forms of acute pancreatitis as the frequency of pancreatic enlargement was very similar to a recent study of cats with presumed chronic pancreatitis with mostly low PLI concentration values.^5^ Also compared to that study,^5^ significantly fewer cats in cluster 1 had a hypoechoic pancreas or a hyperechoic mesentery. The frequency of pancreatic enlargement increased from cluster 1 to 3 from 34% to 75%, likely indicating more acute pancreatitis in clusters 2 and 3. Besides peripancreatic fluid, pancreatic enlargement was the only variable that increased in frequency from cluster 1 to cluster 3. In a recent study in dogs, of all US variables examined, only pancreatic enlargement was identified to increase the likelihood of an acute pancreatitis diagnosis.^24^ Confirming that an enlarged pancreas indicates more acute, clinically relevant pancreatitis in cats requires prospective studies, as pancreatic enlargement in this study reflected subjective radiologist assessment. At present, the pancreas is not routinely measured in the same way as, for example, the adrenal glands. Thus more subtle changes such as mild pancreatic enlargement might be overlooked during US examination. In addition, the pancreas in cats can vary greatly in its total size and volume,^2^ which can make it more difficult to recognize mild increases in size as such. The subjective finding of an ultrasonographically enlarged pancreas was even less common (15% of cats) in an older study in cats with histologically confirmed acute pancreatic necrosis in a study from 2002.^19^ Advanced imaging technology certainly plays a role when comparing results, but it should be noted that subjectively detected pancreatic enlargement is by no means present in all cats with clearly increased lipase levels. When comparing frequencies of pancreatic enlargement and USDx, it becomes clear that it is still unclear when based on which and how many pancreatic abnormalities a radiologist ultimately formulates an USDx. This inconsistency has already been demonstrated once before in cats, albeit with a smaller number of cases.^19^

It is possible that the radiologists did not formulate an USDx in 7 of 12 cats in cluster 3 because of the lack of concurrent US pancreatic changes (ie, a combination of an enlarged, hypoechoic pancreas together with a hyperechoic mesentery) that are more commonly reported in dogs with acute pancreatitis.^24,25^ The limitations of a retrospective study design become clear here. Nevertheless, it is a new finding that in slightly under 60% of cats in cluster 3 with marked hyperlipasemia (LSmean 778 U/L, range 441-1613 U/L) where acute pancreatitis will be suspected, the pancreatic changes on ultrasonography were not adequate for a final USDx. Again, these results are similar to published findings comparing pancreatic US to presence of acute pancreatic necrosis.^19^

Similar to pancreatic enlargement, “peripancreatic fluid” increased from cluster 1 (7%) to 3 (25%) but remained infrequent. Peripancreatic effusion was present in 20% of cats with acute pancreatic necrosis,^19^ but apart from that study very little is known about prevalences of peripancreatic fluid in cats with confirmed pancreatitis. In fact, it is not mentioned in the results section of most available studies.^5,7,18,20^ Three out of four cats with histologically confirmed acute pancreatitis had peripancreatic fluid on ultrasound performed shortly before.^10^ The fact that post mortem examinations were performed suggests that each case must have been severe acute pancreatitis. Similarly, only 17% of cats with suspected pancreatitis and a hypoechoic pancreas had peritoneal effusion in another retrospective study.^17^ Taken together, this suggests that peripancreatic fluid is a rare finding in cats with suspected pancreatitis, potentially indicating that only a small percentage of cats suffer from severe acute pancreatitis. This, in turn, is consistent with the similarly low prevalence of acute pancreatitis documented in histopathological studies.^1–3^

A hyperechoic mesentery together with hyperlipasemia is usually interpreted as reactive steatitis and is usually interpreted as extension of pancreatic inflammation to adjacent fat tissues in cats^20^ and dogs.^26^ A hyperechoic mesentery was the single US characteristic with the highest sensitivity (nevertheless only 68%) to detect pancreatitis in cats.^20^ However, that study equated the presence of pancreatitis with increased PLI concentration values (measured using different assays), which have since been revised. In addition, many cats also had other comorbidities.^20^ A hyperechoic mesentery was present in less than half of cluster 2 cats and only one-third of cluster 3 cats. This was a surprising finding for us, as lipase levels in clusters 2 and 3 were quite high. Our results in cluster 3 cats are similar to a previous finding that only 25% of cats with acute necrotizing pancreatitis (all of which had histologically confirmed peripancreatic fat necrosis) had a hyperechoic mesentery when ultrasounded a median of one day before necropsy.^19^ An ultrasonographically detectable hyperechoic mesentery may only appear later in the course of the disease or only in cases of more severe inflammation of the pancreas in cats. The reason for this is currently unclear. But this is consistent with a recent study in which clinically healthy cats with very high serum lipase activity (up to 283 U/L) and PLI concentration values (up to 86 mcg/L) had at most a hypoechoic and enlarged pancreas but no hyperechoic mesentery.^12^

Frequencies of a hypoechoic and also a mixed-echoic pancreas were significantly different between cluster 2 and cluster 1. Both findings were a little less prevalent in cluster 3 cats which explains the lacking significance versus cluster 1 cats. As a mixed-echoic pancreas includes hypoechoic foci, these 2 findings likely point in the same direction. However, in 50%-60% of cats with marked hyperlipasemia (ie, clusters 2 and 3), the pancreatic tissue was neither hypoechoic nor mixed-echoic in our study (Table 2). These results in clusters 2 and 3 are again only slightly higher compared to a prevalence of 35% for a hypoechoic pancreas in cats with necrotizing pancreatitis.^19^ Poor agreement between a hypoechoic and mixed-echoic pancreas and serum lipase activity and PLI concentration values > RI has been shown before,^10^ but our results indicate that it is obviously no better at markedly elevated lipase activities. Acute pancreatitis in cats is associated with less hemorrhage and edema compared to dogs.^1,19,27^ In addition, necrotic areas were located more in the periphery of the organ in that study.^19^ These 3 factors (hemorrhage, edema, necrosis) contribute to hypoechoic areas in the pancreas, and it is possible that with fewer or different histological findings in feline pancreatitis, the same findings as in dogs cannot be expected.

Regardless of statistical significance after Bonferroni correction, with the exception of pancreatic enlargement, all US findings decreased again in frequency when comparing cluster 2 and cluster 3. The same applies to an USDx and UPASS, which seemed counterintuitive at first glance. It could be assumed that cluster 3 cats were more acutely ill and that there may not have been enough time for pancreatic lesions to show up on US, as has been shown in dogs with acute pancreatitis.^28^ However, disease duration before presentation did not differ between clusters. We have 2 thoughts on this: On the one hand, the secretive nature of cats might play a role here. It is difficult to determine at what time point cats are seriously ill, as even in severe cases they may “only” be lethargic and anorectic. On the other hand, almost all cats had concurrent diseases in addition to pancreatitis, and it is possible that acute pancreatitis developed secondary to an extra-pancreatic disease but the cat had already been “sick” for a while. Biochemical changes (lipase release) might occur more rapidly than structural pancreatic changes. Any condition associated with volume retraction (pancreatic ischemia) and vomiting (retrograde pressure waves facilitating ascending duodenal juice into pancreatic ducts) could trigger a secondary or reactive acute pancreatopathy in an already sick cat. This might even be more likely if unrecognized chronic pancreatitis (which is very common in cats^1^) is present. In a previous study, around 10% of cats without US suspicion of pancreatitis had abnormal PLI concentrations.^7^ Probably more cats with high serum lipase activities without US suspicion of pancreatitis were included in this study because serum lipase activity is included in our routine biochemistry profile whereas external lipase measurement must be requested separately. This situation could change now, as the advertised new DGGR-based pancreatic serum lipase activity test for small animal practice^29^ may allow more hyperlipasemic cats without major changes on US to be identified. Ideally, US pancreatic changes would only be examined in cats without additional diseases, though this is rarely possible as older cats almost always have comorbidities.^8,17,19^

Since a diagnosis of pancreatitis is also based on clinical and laboratory findings besides US findings, we also wanted to examine clusters for differences in clinical and laboratory results. We were aware that these comparisons would be influenced by the concurrent diseases, even though the frequency of comorbidities did not differ significantly between clusters. We were primarily interested in whether abdominal pain, the hallmark sign of acute pancreatitis in humans,^30,31^ would differ between clusters as we assumed that the severity of pancreatitis increases with increasing serum lipase levels. While it is known that cats with pancreatitis rarely display overt signs of abdominal discomfort,^13,16,32^ it remains unclear whether abdominal pain is more frequent in cases with very high lipase values. This motivated our inclusion of clinical signs in the cluster analysis. Although frequencies of abdominal pain increased from cluster 1 to 3, this difference was not significant and overall abdominal pain was still only found in every fourth cluster 3 cat. The only significant difference was that cats in cluster 2 were more lethargic. The reason for this finding remains unclear and may reflect variability in clinician reporting or individual disease course. Ultimately, no consistent pattern emerged to indicate that specific clinical signs became more common with increasing lipase levels. Cats with markedly increased lipase activities (clusters 2 and 3) did not appear to be “clinically distinguishable” than cats in cluster 1. The number of clinical signs was also not significantly different between clusters*.*

Similar to clinical signs, we were aware that the spectrum of comorbidities would make it difficult to clearly distinguish between pancreatitis-associated and non-pancreatitis-associated laboratory changes. It remains unclear whether increases in serum liver enzyme activities or bilirubin concentrations occur more frequently or are more pronounced in cases with varying levels of lipase. Cats in cluster 3 had significantly higher serum urea concentrations while serum creatinine concentrations did not differ. This is suggestive of prerenal azotemia, a well-recognized complication in more severe pancreatitis.^33^ Volume depletion with renal hypoperfusion, inflammation, gastrointestinal bleeding, and a catabolic state may all contribute to increased serum urea concentration in more severe pancreatitis. Azotemia was more frequent in non-survivors in a larger descriptive study on feline pancreatitis,^8^ and pancreatitis and acute kidney injury are also common comorbidities in humans and dogs, each can potentially precipitate the other.^34,35^ We cannot exclude the possibility that a reduced renal function had an effect on lipase activity. A weak association between serum lipase activity and creatinine that was not deemed clinically relevant has been shown in cats before,^36^ however we feel this is unlikely as no correlation was found for any of the 562 cats that had urea and lipase activity measured.^16^ Similarly in dogs, decreased renal excretion during experimental acute kidney injury did not cause consistent and correlated increases in serum PLI concentration and lipase activity.^35^ Cats in cluster 3 had significantly lower protein concentrations while serum albumin concentrations did not differ between clusters which does not support gastrointestinal bleeding secondary to pancreatitis. A delayed hepatic acute-phase response might serve as an explanation. The liver may focus on producing acute-phase reactants over globulins, and albumin may not drop immediately, as it has a longer half-life. Stress-induced plasma cell suppression during severe systemic inflammation can also lead to a more pronounced decrease in globulins than albumin. Unfortunately, serum amyloid A concentrations were not part of the routine chemistry profile at the time of inclusion, therefore this theory cannot be proven. The observation that globulin concentrations are lower in cats with massive hyperlipasemia has not been previously reported in acute pancreatitis and our findings should therefore be verified again in a prospective study.

Triglycerides were significantly higher in cluster 2 compared to cluster 1 and mean triglycerides were still higher in cluster 3 compared to 1. Endocrine disease (mostly diabetes mellitus) was more common in cluster 3 and this may have also contributed to higher triglyceride levels,^37,38^ but the incidence of endocrine disease was the same between clusters 1 and 2, and yet cluster 2 had significantly higher serum triglyceride concentrations. Hypertriglyceridemia is one of the major laboratory abnormalities observed after experimentally induced acute pancreatitis in cats,^39^ and is also found in humans^40^ with acute pancreatitis. In another experimental model of acute pancreatitis in cats, the significant increase in serum cholesterol concentration was likewise presumed to be a consequence of acute pancreatitis.^41^ It is thought that mediators released from the inflamed pancreas inhibit serum lipoprotein lipase activity.^42^ This mechanism has been demonstrated in experimental pancreatitis in rabbits^43^ and might likewise underlie hypertriglyceridemia in cats. Based on our limited observations of normalization of hypertriglyceridemia in the acute setting of pancreatitis, this condition may represent a consequence rather than a cause of the disease; however, this requires confirmation in future studies.

Our study had several limitations. Due to the retrospective design, we relied on medical record findings. Using a cluster-based approach allowed us to bypass the uncertainty of clinical pancreatitis diagnoses, which can vary between clinicians in the assessment of signs such as abdominal pain or lethargy. The same applies to different radiologists. Conversely, however, our results also reflect everyday clinical practice at a referral clinic. Notably, there remains a lack of prospective study on pancreatitis in cats that compares the individual components of the diagnosis. Our study therefore offers a first approach that future studies can build on. Descriptive statistics suggested apparent differences in some instances across the 3 clusters, but these differences were not statistically significant. This may be attributed to unequal cluster sizes and variability within each cluster. Smaller clusters reduce statistical power, making it more difficult to detect significant differences even when absolute values differ. Because of the retrospective design we did not perform an a-priori sample size calculation; instead, we report confidence intervals for the LSmean values of laboratory endpoints so that the size of effects is transparent. Overall, these effect-size estimates suggest that several non-significant findings are more likely due to limited power than to the complete absence of any association.

In summary, our results show that even with markedly abnormal lipase activities, in many cats pancreatic abnormalities are not detected ultrasonographically or detected changes are not enough to lead to an USDx. Our results are similar to a study using pancreatic histology as the diagnostic gold standard.^19^ Slightly better performance of US for the diagnosis of pancreatitis in our study could be mainly due to better technology and image resolution, but the core findings and unanswered questions remain the same. Routine determination of pancreatic size might help in the future. Our findings further suggest that clinical signs remain nonspecific in sick cats, irrespective of serum lipase levels. Urea, protein, and triglyceride levels might help estimate the severity or acuteness of pancreatitis in cats; however, these differences might also be influenced by concurrent conditions or other confounding factors.

## Supplementary Material

aalag072_Supplemental_Files

## Data Availability

The data underlying this article are available in the article and in its online supplementary material.

## Off-label antimicrobial declaration

Authors declare no off-label use of antimicrobials.

## Institutional animal care and use committee or other approval declaration

Authors declare no institutional animal care and use committee or other approval was needed.

## Appendix

### Statistical analyses

We applied k-means cluster analysis to the raw serum lipase activities using the procedures HPCLUS and FASTCLUS of SAS 9.4 (SAS Institute Inc.). Candidate solutions with k = 2-14 clusters were evaluated. The optimal number of clusters was assessed using the gap statistic (ABC statistics plot based on within-cluster sum of squares). The gap statistic showed a clear maximum at k = 2 and a local maximum at k = 3; according to the 1-SE rule, both solutions were supported, and we chose k = 3 as a parsimonious and clinically interpretable model (Figure S1). We therefore retained 3 clusters that corresponded to low, moderately abnormal, and markedly abnormal serum lipase activities (Figure S1, Table S1).

As sensitivity analyses, we explored alternative clustering approaches (hierarchical clustering with Ward’s linkage and Gaussian mixture modeling with 3 components). These methods yielded almost identical partitions of the data and the same pattern of associations with clinical, laboratory, and ultrasonographic variables (data not shown). We additionally fitted multivariable general linear models in which serum lipase, age and body weight were entered as continuous predictors instead of the cluster factor. These models produced effect estimates and *P*-values that did not materially differ from the cluster-based analyses (data not shown). To avoid overfitting and to provide a clinically easier-to-interpret presentation, we therefore report the cluster-based models as our primary analysis.

Age, body weight, duration of disease before presentation, duration of hospitalization (DoH), duration of intensive care unit stay (DoIPS) and laboratory variables were analyzed using general linear models (PROC GLM in SAS 9.4) including the fixed effects “cluster” (1-3) and “sex” (male, female, male-castrated, female-spayed). Initial models also contained a cluster×sex interaction term; because these interactions were not statistically significant and difficult to interpret given the small subgroup sizes, they were removed from the final models, which therefore present main effects only. Model assumptions (normality and homoscedasticity of residuals) were assessed by visual inspection of residual-versus-fitted plots and Q–Q plots, and by Shapiro–Wilk and Levene tests; no major violations were detected. Tukey’s honest significance difference test was used for post-hoc pairwise comparisons of LSmeans while controlling the family-wise error rate at α ≤ 0.05. In Table S2, we report effects sizes based on LSmeans with Tukey-adjusted 95% CI, and exact Tukey-adjusted *P*-values.

Binary clinical and ultrasonographic variables were analyzed by Fisher’s exact tests with cluster as grouping factor. For each variable, we first conducted an overall 3 × 2 test, followed by 3 pre-specified pairwise comparisons between clusters (1 vs 2, 1 vs 3, and 2 vs 3). To control the Type I error rate for these 3 comparisons, we applied a Bonferroni correction and considered *P* ≤ .05/3 = .0167 statistically significant. Given the limited event counts in some cells, we deliberately chose this conservative approach to reduce the risk of false-positive findings and over-interpretation of results from this retrospective clinical cohort. Exact 2-sided *P*-values are reported in Table S3.

All statistical analyses were conducted in SAS 9.4. The complete SAS code and all relevant HPCLUS/FASTCLUS and GLM options are provided as Supplementary Material to facilitate reproducibility.

## Abbreviations

DGGR: 1,2-o-dilauryl-rac-glycero-3-glutaric acid-(6 0-methylresorufin) ester
USDx: ultrasonographic diagnosis of pamcreatitis
US: ultrasonography 7 ultrasonographic
UPASS: ultrasonographic pancreatic assessment severity score
DHo: duration of hospitalization
DoICU: duration of intensive care unit stay
