# Supplementary material for: Unsupervised clustering of serum lipase activity in cats: a data-driven approach to correlate clinical, laboratory, and ultrasonographic findings
Source: J Vet Intern Med. 2026 Apr 21;40(2):aalag072. doi: 10.1093/jvimsj/aalag072 (PMC13098366; doi:10.1093/jvimsj/aalag072)
Supplement: aalag072_Supplemental_Files [file aalag072_supplemental_files.zip › Supplementary_Figure_S1_aalag072.docx]

**Supplementary Figure S1.***ABC statistics (“gap” values) for determining the optimal number of k-means clusters based on serum lipase activity.*

**
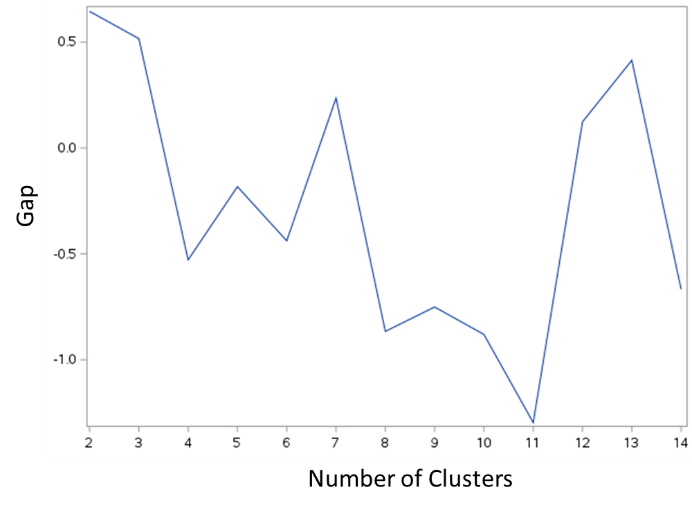
**

*Note: Gap values from the Aligned Box Criterion (ABC) are plotted against the number of clusters (k) for k = 2–14 in 563 cats. The curve shows the highest gap value at k = 2 and a clear local maximum at k = 3; according to the 1-standard-error rule, both k = 2 and k = 3 are plausible solutions, but we considered k = 3 as the optimal and clinically most interpretable choice (low, intermediate, and high serum lipase activity), and used this 3-cluster solution in all primary analyses.*
