# Supplementary material for: Unsupervised clustering of serum lipase activity in cats: a data-driven approach to correlate clinical, laboratory, and ultrasonographic findings
Source: J Vet Intern Med. 2026 Apr 21;40(2):aalag072. doi: 10.1093/jvimsj/aalag072 (PMC13098366; doi:10.1093/jvimsj/aalag072)
Supplement: aalag072_Supplemental_Files [file aalag072_supplemental_files.zip › Supplementary_Table 4_Cluster_3_Diagnoses_aalag072.docx]

**Supplementary Table 4: Clinical diagnoses of cluster 3 cats.**

| **Cat**  **(n=15)** | **Comorbidities** |
| --- | --- |
| 1 | Spinal neoplasia, diabetes mellitus |
| 2 | Chronic kidney disease |
| 3 | Diabetic ketoacidosis, chronic enteropathy |
| 4 | Cholecystitis, cholelithiasis with secondary cholestasis and acute kidney injury |
| 5 | Neoplastic pleural effusion (cytologically suspicion of carcinoma) |
| 6 | Peritoneal carcinomatosis, mediastinal mass |
| 7 | Septic peritonitis |
| 8 | Chronic kidney disease, pyelonephritis, chronic enteropathy |
| 9 | Diabetes mellitus |
| 10 | Diabetes mellitus |
| 11 | Large cell lymphoma |
| 12 | Hepatic lipidosis, multiple hepatic masses |
| 13 | Septic peritonitis |
| 14 | Oral cavity carcinoma |
| 15 | Diabetes mellitus |

*Note: The table summarizes clinical diagnoses in 15 cats within Cluster 3.*
