# Supplementary material for: Unsupervised clustering of serum lipase activity in cats: a data-driven approach to correlate clinical, laboratory, and ultrasonographic findings
Source: J Vet Intern Med. 2026 Apr 21;40(2):aalag072. doi: 10.1093/jvimsj/aalag072 (PMC13098366; doi:10.1093/jvimsj/aalag072)
Supplement: aalag072_Supplemental_Files [file aalag072_supplemental_files.zip › Supplementary_Tables_S1-S3_aalag072.docx]

**Supplementary Table 1:** *Aligned Box Criterion (ABC) statistics for k-means clustering of serum lipase activity in 563 cats.*

| **Number of clusters (k)** | **Log within-cluster SSE (input)** | **Log within-cluster SSE (reference)** | **Gap value** | **Simulation-corrected SD** | **One-SE-corrected gap** |
| --- | --- | --- | --- | --- | --- |
| 2 | 15.2039 | 15.8493 | 0.6454 | 0.0602 | 0.5852 |
| 3 | 14.5646 | 15.0814 | 0.5168 | 0.2517 | 0.2650 |
| 4 | 14.1809 | 13.6526 | –0.5283 | 0.1520 | –0.6803 |
| 5 | 14.0781 | 13.8954 | –0.1827 | 0.4847 | –0.6674 |
| 6 | 14.0411 | 13.6028 | –0.4383 | 0.6002 | –1.0386 |
| 7 | 14.0295 | 14.2651 | 0.2356 | 0.4937 | –0.2582 |
| 8 | 13.9903 | 13.1241 | –0.8662 | 0.1569 | –1.0231 |
| 9 | 13.9895 | 13.2385 | –0.7510 | 0.2461 | –0.9971 |
| 10 | 13.9893 | 13.1086 | –0.8807 | 0.3464 | –1.2271 |
| 11 | 13.8687 | 12.5716 | –1.2971 | 0.2752 | –1.5724 |
| 12 | 13.8622 | 13.9859 | 0.1237 | 0.3800 | –0.2563 |
| 13 | 13.8622 | 14.2766 | 0.4144 | 0.2345 | 0.1799 |
| 14 | 13.8589 | 13.1921 | –0.6668 | 0.7043 | –1.3711 |

*Note: Gap values and associated statistics were obtained from the SAS HPCLUS procedure using the Aligned Box Criterion with 10 reference distributions and PCA alignment. The one-standard-error–corrected gap is defined as Gap(k) – s k, where s k is the simulation-corrected standard deviation. According to the 1-SE rule, a solution with k = 2–3 clusters is supported; we selected k = 3 as the optimal, clinically interpretable solution and used this in all subsequent models.*

**Supplementary Table S2.** *Cluster effect sizes expressed as LSmeans with 95% Tukey-adjusted confidences limits and Tukey-adjusted between cluster p-values and the global effect p-value*

|  | **LSmeans and 95%**  **Confidence Limits (Cl)** | | | **Tukey-adjusted P** | | |  |
| --- | --- | --- | --- | --- | --- | --- | --- |
|  | **Cluster 1** | **Cluster 2** | **Cluster 3** | **Cluster 1 vs. 2** | **Cluster 1 vs. 3** | **Cluster 2 vs. 3** | **global P Cluster** |
| Age, years | 8.84 Cl  [8.07, 9.63] | 8.17 Cl  [6.80, 9.55] | 9.37 Cl  [7.02, 11.72] | 0.53 | 0.89 | 0.62 | 0.49 |
| Weight, kg | 4.26 Cl  [3.91, 4.62] | 4.15 Cl  [3.53, 4.77] | 4.29 Cl  [3.24, 5.35] | 0.91 | 1.00 | 0.96 | 0.92 |
| DoI, days | 15.7 Cl  [-0.36, 31.7] | 11.7 Cl  [-16.5, 40.0] | 1.62 Cl  [-47.7, 51.0] | 0.95 | 0.83 | 0.92 | 0.81 |
| DoH, days | 3.65 C  l[3.14, 4.17] | 3.42 Cl  [2.51, 4.32] | 3.75 Cl  [2.19, 5.31] | 0.83 | 0.99 | 0.92 | 0.84 |
| DoIPS, days | 1.75 Cl  [1.41, 2.10] | 1.47 Cl  [0.86, 2.08] | 2.25 Cl  [1.21, 3.30] | 0.57 | 0.6 | 0.36 | 0.35 |
| Hematocrit, % | 32.9 Cl  [29.0, 36.7] | 31.8 Cl  [25.2, 38.4] | 26.6 Cl  [15.1, 38.2] | 0.94 | 0.51 | 0.69 | 0.52 |
| Leucocytes, 103/mcl | 14.7 Cl  [12.3, 17.0] | 16.6 Cl  [12.5, 20.6] | 13.9 Cl  [6.80, 21.0] | 0.55 | 0.97 | 0.76 | 0.56 |
| Thrombocytes, 103/mcl | 281 Cl  [246, 316] | 276 Cl  [220, 332] | 282 Cl  [182, 381] | 0.97 | 0.99 | 0.99 | 0.98 |
| Band Neutrophils, 103/mcl | 9.11 Cl  [-1.11, 19.33] | 5.31 Cl  [-12.0, 22.6] | 5.11 Cl  [-23.4, 33.6] | 0.88 | 0.95 | 0.99 | 0.86 |
| Segmented Neutrophils, 103/mcl | 11.5 Cl  [9.49, 13.56] | 15.3 Cl  [11.8, 18.7] | 10.7 Cl  [5.07, 16.3] | 0.05 | 0.95 | 0.3 | 0.05 |
| Lymphocytes, 103/mcl | 1.86 Cl  [1.43, 2.29] | 2.00 Cl  [1.30, 2.71] | 1.28 Cl  [0.07, 2.49] | 0.89 | 0.58 | 0.5 | 0.54 |
| Bilirubin, mmol/l | 14.5 Cl  [8.13, 21.0] | 13.7 Cl  [2.56, 24.9] | 14.2 Cl  [-5.21, 33.7] | 0.99 | 0.99 | 0.99 | 0.99 |
| Glucose, mmol/l | 9.37 Cl  [4.46, 14.3] | 7.98 Cl  [-0.58, 16.5] | 15.2 Cl  [-0.08, 30.5] | 0.93 | 0.72 | 0.66 | 0.68 |
| Urea,  mmol/l | 16.3 Cl  [8.62, 24.0] | 16.7 Cl  [3.44, 29.9] | 64.1 Cl  [39.3, 88.9] | 0.99 | 0.0003 | 0.001 | 0.0005 |
| Creatinine, mcmol/l | 185 Cl  [138, 232] | 212 Cl  [133, 292] | 223 Cl  [74.1, 372] | 0.73 | 0.86 | 0.99 | 0.67 |
| Total protein, g/l | 67.0 Cl  [64.9, 69.0] | 68.6 Cl  [65.0, 72.2] | 59.2 Cl  [52.5, 65.9] | 0.59 | 0.04 | 0.03 | 0.03 |
| Albumin,  g/l | 31.8 Cl  [28.3, 35.3] | 31.4 Cl  [25.3, 37.5] | 27.8 Cl  [16.8, 38.9] | 0.98 | 0.75 | 0.83 | 0.76 |
| Cholesterol, mmol/l | 4.00 Cl  [3.41, 4.60] | 3.90 Cl  [2.86, 4.94] | 4.34 Cl  [2.35, 6.32] | 0.97 | 0.93 | 0.91 | 0.92 |
| Triglycerides, mmol/l | 1.81 Cl  [0.83, 2.79] | 3.75 Cl  [2.04, 5.46] | 2.74 Cl  [-0.53, 6.01] | 0.04 | 0.83 | 0.83 | 0.04 |
| Alkaline Phosphatase, U/l | 50.8 Cl  [35.1, 66.4] | 47.3 Cl  [19.9, 74.8] | 37.4 Cl  [-15.4, 90.3] | 0.96 | 0.86 | 0.93 | 0.85 |
| Lipase, U/l | 52.9 Cl  [40.9, 64.9] | 229 Cl  [208, 251] | 778 Cl  [742, 814] | <0.0001 | <0.0001 | <0.0001 | <0.0001 |
| ASAT, U/l | 503 Cl  [292, 715] | 909 Cl  [534, 1284] | 430 Cl  [-296, 1156] | 0.04 | 0.98 | 0.43 | 0.05 |
| ALAT, U/l | 543 Cl  [433, 653] | 634 Cl  [441, 828] | 546 Cl  [184, 908] | 0.55 | 0.99 | 0.89 | 0.58 |
| Ca, mmol/l | 2.46 Cl  [1.52, 3.40] | 2.13 Cl  [0.50, 3.76] | 2.08 Cl  [-0.95, 5.11] | 0.90 | 0.96 | 0.99 | 0.88 |
| SAA, mg/l | 65.9 Cl  [37.9, 94.0] | 79.3 Cl  [23.1, 144] | 66.7 Cl  [-40.1, 173] | 0.9 | 0.99 | 0.98 | 0.91 |
| Urine-specific Weight | 1028 Cl  [1019, 1038] | 1024 Cl  [1012, 1037] | 1028 Cl  [1001, 1056] | 0.67 | 1.00 | 0.96 | 0.7 |

**Supplementary Table S3.** *Between-cluster p-values and global p-values of ultrasonographic (US) findings and clinical signs from a two-tailed Fisher's-Exact Test (Bonferroni threshold P ≤ 0.0167)*

|  |  | **Between-cluster P** | | |  |
| --- | --- | --- | --- | --- | --- |
|  |  | **Cluster**  **1 vs. 2** | **Cluster**  **1 vs. 3** | **Cluster**  **2 vs. 3** | **global P Cluster** |
| Pancreas | US performed | 0.0136 | 0.0636 | 0.7429 | 0.0057 |
|  | USDx | 0.0021 | 0.5494 | 0.5571 | 0.0055 |
|  | UPASS | 0.0002 | 0.0212 | 0.9374 | 0.0011 |
|  | Enlargement | 0.0041 | 0.01 | 0.4967 | 0.0003 |
|  | Hypoechogenicity | 0.0071 | 0.5458 | 0.5113 | 0.0193 |
|  | Hyperechogenicity | 0.8148 | 0.6991 | 0.6618 | 0.7897 |
|  | Mixed echogenicity | 0.0072 | 0.1894 | 1 | 0.0106 |
|  | Hyperechoic mesentery | 0.0017 | 0.4904 | 0.5095 | 0.0039 |
|  | Peripancreatic fluid | 0.2082 | 0.0588 | 0.372 | 0.0438 |
| Hepato-  biliary  system | Gallbladder sludge | 0.5359 | 0.7174 | 1 | 0.7204 |
|  | Enlargement | 0.8601 | 0.7604 | 0.7308 | 0.874 |
|  | Hypoechogenicity | 0.1347 | 0.3826 | 1 | 0.1382 |
|  | Hyperechogenicity | 1.0000 | 0.5556 | 0.7402 | 0.8002 |
|  | Mixed echogenicity | 0.8219 | 0.1281 | 0.2527 | 0.2814 |
| Intestines | L. muscularis thickening | 0.8629 | 0.1382 | 0.1741 | 0.2942 |
|  | Mesenteric lymphadenopathy | 0.2222 | 0.5545 | 0.3184 | 0.3385 |
|  | Intestinal mass | 1.0000 | 0.0112 | 0.0343 | 0.0158 |
| Clinical Signs | Number of clinical signs | 0.1554 | 0.2504 | 0.3783 | 0.1644 |
|  | Vomiting | 0.0397 | 0.5939 | 0.772 | 0.0813 |
|  | Bloody vomiting | 0.5664 | 0.2141 | 0.39 | 0.1711 |
|  | Diarrhoea | 0.5495 | 0.7091 | 0.4343 | 0.5716 |
|  | Bloody diarrhoea | 1.0000 | 1 | 1 | 1 |
|  | Lethargy | 0.0093 | 0.2986 | 0.7614 | 0.014 |
|  | Anorexia | 0.2529 | 0.2962 | 0.7672 | 0.2649 |
|  | Painful abdomen | 0.4911 | 0.0674 | 0.2374 | 0.0998 |
|  | Fever | 0.5194 | 0.5637 | 1 | 0.5414 |
|  | Icterus | 1.0000 | 1 | 1 | 1 |
|  | Pu/Pd | 0.8440 | 0.2752 | 0.4582 | 0.4491 |
|  | Weight loss | 0.2321 | 0.3443 | 0.7614 | 0.2461 |
| Comorbidities | Gastrointestinal disease | 0.7374 | 0.2103 | 0.1622 | 0.3169 |
|  | Nephrology/urology disease | 0.7335 | 0.54 | 0.4942 | 0.7121 |
|  | Hepatobiliary disease | 1.0000 | 1 | 1 | 1 |
|  | Endocrine disease | 0.6842 | 0.0579 | 0.1665 | 0.1219 |
|  | Cardiac disease | 0.5432 | 0.241 | 0.1891 | 0.2463 |
|  | Respiratory disease | 0.0496 | 0.6365 | 1 | 0.0652 |
|  | Musculoskeletal disease | 0.0483 | 1 | 0.39 | 0.089 |
|  | Nervous system | 1.0000 | 0.6147 | 0.5696 | 0.8359 |
|  | Ophthalmologic disease | 0.0613 | 1 | 0.2174 | 0.1223 |
