# Supplementary material for: Unsupervised clustering of serum lipase activity in cats: a data-driven approach to correlate clinical, laboratory, and ultrasonographic findings
Source: J Vet Intern Med. 2026 Apr 21;40(2):aalag072. doi: 10.1093/jvimsj/aalag072 (PMC13098366; doi:10.1093/jvimsj/aalag072)
Supplement: aalag072_Supplemental_Files [file aalag072_supplemental_files.zip › Supplementary_Text_S1_aalag072.docx]

**Supplementary Text S1:** SAS code used for clustering and GLM analyses

***K-means cluster analysis:***

*proc hpclus data=lipase maxclusters=14*

*NOC=ABC(B=10 minclusters=2 align=PCA criterion=FIRSTPEAK);*

*input  Lipase;*

*ods output ABCStats=ABCStats1;*

*run;*

*title "ABC Statistics Plot Using Gap Values";*

*proc sgplot data=ABCStats1;*

*xaxis type=discrete;*

*series x= K y= Gap;*

*run;*

*title ' ';*

*quit;*

*proc fastclus data=lipase maxc=3 maxiter=10 out=clus;*

*var Lipase;*

*run;*

*proc freq;*

*tables cluster*Tier/nopercent norow nocol;*

*run;*

*proc candisc anova out=can;*

*class cluster;*

*var Lipase;*

*title2 'Canonical Discriminant Analysis of Macrophage Gene Expression';*

*run;*

*proc sgplot data=Can;*

*scatter x=Cluster y=Lipase / group=Cluster;*

*title2 'Plot of Canonical Variables Identified by Cluster';*

*run;*

***GLM and Normality via Proc Univariate:***

*ods graphics on;*

*proc glm ;*

*class Tier sex Cluster ;*

*model Alter Gewicht DoI DoH DoIPS hematocrit leukocytes Thrombocytes band neutrophils segmented neutrophils Lymphs Bilirubin Glucose Urea Creatinin Total protein Albumin Cholesterol Triglyzeride alkaline phosphatase Lipase ASAT ALAT Ca Vit B12 SAA urine specific weight = Cluster sex /ss3 solution clparm;*

*lsmeans cluster/adjust=tukey;*

*output out=outRes  r=r Alter r Gewicht r DoI r DoH r DoIPS r hematocrit r leukocytes r Thrombocytes r band neutrophils r segmented neutrophils r Lymphs r Bilirubin r Glucose r Urea r Creatinin r Total protein r Albumin r Cholesterol r Triglyzeride r alkaline phosphatase r Lipase r ASAT r ALAT r Ca r Vit B12 r SAA r urine specific weight ;*

*run;*

*quit;*

*proc univariate data=outRes normal noprint;*

*var r Alter r Gewicht r DoI r DoH r DoIPS r hematocrit r leukocytes r Thrombocytes r band neutrophils r segmented neutrophils r Lymphs r Bilirubin r Glucose r Urea r Creatinin r Total protein r Albumin r Cholesterol r Triglyzeride r alkaline phosphatase r Lipase r ASAT r ALAT r Ca r Vit B12 r SAA r urine specific weight ;  /* the residual variable created above */*

*qqplot r Alter r Gewicht r DoI r DoH r DoIPS r hematocrit r leukocytes r Thrombocytes r band neutrophils r segmented neutrophils r Lymphs r Bilirubin r Glucose r Urea r Creatinin r Total protein r Albumin r Cholesterol r Triglyzeride r alkaline phosphatase r Lipase r ASAT r ALAT r Ca r Vit B12 r SAA r urine specific weight /*

*normal(mu=est sigma=est)*

*square;*

*run;*

*quit;*

*ods graphics off;*

***Fishers Exact Tests (Overall and between Clusters):***

***Overall Fisher***

*proc freq data=cluster;*

*tables Cluster*done Cluster*enlargement Cluster*hypoechogenicity Cluster*hyperechogenicity*

*Cluster*mixed echogenicity Cluster*reactive mesentery Cluster*fluid peripancreatic Cluster*clear diagnosis*

*Cluster*vague statement Cluster*clearorvague Cluster*done Cluster*sludge Cluster*enlargement*

*Cluster*hypoechogenicity Cluster*hyperechogenicity Cluster*mixed echogenicity Cluster*done*

*Cluster*L muscularis hypertrophie Cluster*lk hypertrophie Cluster*mass Cluster*US triaditis*

*Cluster*biopsyp done Cluster*sectio done Cluster*vomiting Cluster*bloody vomiting Cluster*diarrhoea*

*Cluster*bloody diarrhoea Cluster*apathy Cluster*anorexia Cluster*painful abdomen Cluster*fever*

*Cluster*icterus Cluster*PU PD Cluster*weight loss Cluster*Pancreatopathy Cluster*gastrointestinal disease*

*Cluster*renal urinary disease Cluster*hepatobiliary disease Cluster*endocrine disease Cluster*cardiac disease*

*Cluster*respiratory disease Cluster*musculoskeletal disease Cluster*h Cluster*immune mediated disease*

*Cluster*neoplastic disease Cluster*ophthalmologic disease Cluster*Reproductive system Cluster*USDx*

*Cluster*previous steroid /fisher;*

*run;*

*Quit;*

***Cluster 1 vs. 2***

*proc freq data=cluster(where=(cluster in (1,2)));*

*tables Cluster*done Cluster*enlargement Cluster*hypoechogenicity Cluster*hyperechogenicity*

*Cluster*mixed echogenicity Cluster*reactive mesentery Cluster*fluid peripancreatic Cluster*clear diagnosis*

*Cluster*vague statement Cluster*clearorvague Cluster*done Cluster*sludge Cluster*enlargement*

*Cluster*hypoechogenicity Cluster*hyperechogenicity Cluster*mixed echogenicity Cluster*done*

*Cluster*L muscularis hypertrophie Cluster*lk hypertrophie Cluster*mass Cluster*US triaditis*

*Cluster*biopsyp done Cluster*sectio done Cluster*vomiting Cluster*bloody vomiting Cluster*diarrhoea*

*Cluster*bloody diarrhoea Cluster*apathy Cluster*anorexia Cluster*painful abdomen Cluster*fever*

*Cluster*icterus Cluster*PU PD Cluster*weight loss Cluster*Pancreatopathy Cluster*gastrointestinal disease*

*Cluster*renal urinary disease Cluster*hepatobiliary disease Cluster*endocrine disease Cluster*cardiac disease*

*Cluster*respiratory disease Cluster*musculoskeletal disease Cluster*h Cluster*immune mediated disease*

*Cluster*neoplastic disease Cluster*ophthalmologic disease Cluster*Reproductive system Cluster*USDx*

*Cluster*previous steroid /fisher;*

*run;*

*quit;*

***Cluster 1 vs. 3***

*proc freq data=cluster(where=(cluster in (1,3)));*

*tables Cluster*done Cluster*enlargement Cluster*hypoechogenicity Cluster*hyperechogenicity*

*Cluster*mixed echogenicity Cluster*reactive mesentery Cluster*fluid peripancreatic Cluster*clear diagnosis*

*Cluster*vague statement Cluster*clearorvague Cluster*done Cluster*sludge Cluster*enlargement*

*Cluster*hypoechogenicity Cluster*hyperechogenicity Cluster*mixed echogenicity Cluster*done*

*Cluster*L muscularis hypertrophie Cluster*lk hypertrophie Cluster*mass Cluster*US triaditis*

*Cluster*biopsyp done Cluster*sectio done Cluster*vomiting Cluster*bloody vomiting Cluster*diarrhoea*

*Cluster*bloody diarrhoea Cluster*apathy Cluster*anorexia Cluster*painful abdomen Cluster*fever*

*Cluster*icterus Cluster*PU PD Cluster*weight loss Cluster*Pancreatopathy Cluster*gastrointestinal disease*

*Cluster*renal urinary disease Cluster*hepatobiliary disease Cluster*endocrine disease Cluster*cardiac disease*

*Cluster*respiratory disease Cluster*musculoskeletal disease Cluster*h Cluster*immune mediated disease*

*Cluster*neoplastic disease Cluster*ophthalmologic disease Cluster*Reproductive system Cluster*USDx*

*Cluster*previous steroid /fisher;*

*run;*

*quit;*

***Cluster 2 vs. 3***

*proc freq data=cluster(where=(cluster in (2,3)));*

*tables Cluster*done Cluster*enlargement Cluster*hypoechogenicity Cluster*hyperechogenicity*

*Cluster*mixed echogenicity Cluster*reactive mesentery Cluster*fluid peripancreatic Cluster*clear diagnosis*

*Cluster*vague statement Cluster*clearorvague Cluster*done Cluster*sludge Cluster*enlargement*

*Cluster*hypoechogenicity Cluster*hyperechogenicity Cluster*mixed echogenicity Cluster*done*

*Cluster*L muscularis hypertrophie Cluster*lk hypertrophie Cluster*mass Cluster*US triaditis*

*Cluster*biopsyp done Cluster*sectio done Cluster*vomiting Cluster*bloody vomiting Cluster*diarrhoea*

*Cluster*bloody diarrhoea Cluster*apathy Cluster*anorexia Cluster*painful abdomen Cluster*fever*

*Cluster*icterus Cluster*PU PD Cluster*weight loss Cluster*Pancreatopathy Cluster*gastrointestinal disease*

*Cluster*renal urinary disease Cluster*hepatobiliary disease Cluster*endocrine disease Cluster*cardiac disease*

*Cluster*respiratory disease Cluster*musculoskeletal disease Cluster*h Cluster*immune mediated disease*

*Cluster*neoplastic disease Cluster*ophthalmologic disease Cluster*Reproductive system Cluster*USDx*

*Cluster*previous steroid /fisher;*

*run;*

*quit;*
